# Effect of print parameters on additive manufacturing of metallic parts: performance and sustainability aspects

**DOI:** 10.1038/s41598-022-22613-2

**Published:** 2022-11-11

**Authors:** Thongchai Fongsamootr, Itthidet Thawon, Nakorn Tippayawong, Korrakot Yaibuathet Tippayawong, Pana Suttakul

**Affiliations:** 1 Department of Mechanical Engineering, Faculty of Engineering, Chiang Mai University, Chiang Mai, 50200 Thailand; 2 Department of Industrial Engineering, Faculty of Engineering, Chiang Mai University, Chiang Mai, 50200 Thailand; 3 Supply Chain and Engineering Management Research Unit, Chiang Mai University, Chiang Mai, 50200 Thailand

**Keywords:** Mechanical engineering, Mechanical properties, Design, synthesis and processing

## Abstract

In this study, the effects of print parameters on the mechanical properties of additively manufactured metallic parts were investigated using a tensile test. The 17-4 PH stainless steel specimens with two print parameters, including infill density and pattern orientation, were fabricated by additive manufacturing (AM) using the bound metal deposition (BMD) technique. The mechanical properties considered in this study are the Young’s modulus and ultimate tensile strength. The results demonstrate that the pattern orientations do not affect the Young’s modulus of the infill specimen with the triangular pattern. In contrast, the ultimate strength significantly varies depending on the pattern orientations, where the samples with the pattern orientation of zero degrees yield the best ultimate strength. In fact, the mechanical properties of infill specimens increase with their infill density. However, when operating cost and time are considered, an index for estimating performance and sustainability is consequently established. The relationship between the normalized ultimate strength of an infill specimen and the relative density is defined as the weight efficiency. The index for assessing a sustainable product is characterized by the weight efficiency versus sustainable parameter(s). The index can help end users select an appropriate infill density for AM products by considering the operating cost and time. Different cost models, including material-only costs, direct costs, and total costs, can be included in the index model to assess a sustainable product in a particular cost context.

## Introduction

The proliferation of human development in the twenty-first century involves a fundamental change across technological revolution and human life, known as the Fourth Industrial Revolution or Industry 4.0. Civilization is on the brink of a new industrial revolution to increase global industrial performance and improve quality of life worldwide, driven by modern manufacturing technologies and information systems. A new generation of manufacturing systems can provide optimal processes and technologies, i.e., artificial intelligence, robotics, the internet of things, and additive manufacturing (AM), for flexible production with long-term gains in efficiency and productivity^1–3^. Among the technological advances, AM is regarded as a crucial manufacturing process driving Industry 4.0. AM is a manufacturing technique to fabricate parts from a three-dimensional (3D) computer-aided design (CAD) file, also known as 3D printing. It effortlessly enables fabrication of complex parts with freeform shapes by a layer-by-layer process^4^. This technique has less waste material in the manufacturing process than traditional subtractive processes, e.g., computer numeric control (CNC) machines, waterjets, and laser cutting. Since AM parts can be designed with greater shape freedom and less manufacturing waste, AM can be applied as a novel manufacturing process for designable and customizable products, potentially creating a new business model.

In addition to being a driving technology of Industry 4.0, AM can be regarded as a sustainable technology for the environment since the technology provides less waste material and can use recycled material in manufacturing. It is a productive process that can considerably reduce global greenhouse gas emissions. Massive raw material production in industrial sectors and their potential environmental footprint can potentially be reduced using AM technologies. In that case, AM may provide opportunities for increasing environmental sustainability in various industries. Formerly, sustainability was defined by emphasizing the environmental aspect. Currently, the definition also includes societal and economic aspects, which AM technology also addresses^5^. Referring to the 6R principles (reduce, recover, recycle, reuse, redesign, and remanufactured)^6^, AM offers a possibility for sustainable development, bettering the social and economic impacts of the whole product life cycle^7,8^. AM allows simultaneous development of environmental conservation and economic growth, thus optimizing consumption and conserving resources for human life. In light of the mechanics of economic development, a circular economy and the use of resources are substantially related. If material and energy consumption in industries can be reduced and controlled, traditional business models can soon be transformed into sustainable circular economy models^1,9^.

AM technology began in the 1980s as a technique for creating prototypes. Over the years, several AM techniques have been proposed for appropriate applications and user-friendly use. These advances give AM more advantages in manufacturing, such as easier prototyping, no required special tools and skills, potential for massive customized productions, reduced time and cost, and sustainable production^8,10^. Additionally, with the ability for on-demand manufacturing, AM can shorten supply chains, reduce storage needs and delivery costs, and provide a shorter lead-time for replacement parts^11^. These benefits make AM a modern and more accessible manufacturing and logistics process, especially for small businesses with innovative products designed themselves. For large-scale businesses, AM technology has been extensively adopted for various applications, e.g., in aircraft, aerospace, automotive, biomedical, and electronics industries^12–16^. Numerous material types, e.g., polymers, ceramics, and metals, can be used in its production. With continued innovations of AM technologies and materials and the application in various productions and industrial markets, investment in AM has grown significantly in the last half-decade. After the COVID-19 pandemic somewhat subsided, many industries entered a period of advancement and investment. According to Wohlers Report 2022^17^, the AM industry grew by 19.5% in 2021, increasing from 7.5% growth in 2020, the beginning of the COVID-19 outbreak. Moreover, AMPOWER, the leading consultancy in the AM industrial field, has predicted that the AM market will have a yearly growth of 18.2% over the next four years. This report implies that the overall 3D printing sector will be worth approximately 20 billion euros by 2026^18^.

Among AM materials, metals have received more attention from researchers and industries in numerous fields. Metal AM has been forecasted to achieve annual growth of over 29% from 2021 to 2025^19^. A conventional AM technique for metal parts is powder bed fusion (PBF), which spreads powder to form a metal part fused selectively by a high-energy beam. However, this technique requires strict safety regulations and is an expensive operation. A recent alternative metal AM method is the extrusion-based technique based on the fused filament fabrication (FFF) process, in which the fused filaments are mixed from polymer, binder, and powder of a material. Desktop Metal Inc. (DM) has proposed an extrusion-based metal AM technique, called bound metal deposition (BMD), by replacing the traditional raw material with metal, such as stainless steel, copper, and titanium, in the filaments.

The BMD technique enables end-user producers to customize their metal parts. With densities and feature accuracy similar to casting, BMD can manufacture difficult-to-machine parts featuring complex geometry. It can also customize metal parts to have infill or fully dense features depending on the requirements of strength and weight. Although their products have certain drawbacks, i.e., porosity^20^ and surface roughness, the BMD provides fewer steps, effortlessly removable supports, and a user-friendly user interface with step-by-step guidance. Consequently, this technique makes producing metal parts more accessible and safer with no loose metal powders or lasers and less operator intervention. In the printing process, there are two extruders containing metal rods and ceramic rods for forming a metal 3D object and ceramic support media, respectively. After that, the binder is dissolved in the debinding process and densified in the sintering process^21,22^. In terms of using AM parts in engineering applications, the mechanical performance of AM parts can be varied by changing print parameters (e.g., infill density and printing orientation). If a certain strength is required of the part for an application, the appropriate setting of such parameters should be considered. Otherwise, a part with a higher strength than the requirement unnecessarily impacts manufacturing time and costs. Consequently, optimizing strength and manufacturing costs of an AM part is significant. From a sustainability point of view, the part should have optimized strength coupled with reasonable production cost and time.

Over the last few years, although many aspects of AM product behavior have been widely studied and published by many researchers^21,23–28^, AM using the BMD technique has not been explored sufficiently, especially in terms of sustainability. As mentioned earlier, BMD technology enables low volume production and economical metal parts with more safety in the manufacturing process, benefiting small companies, inventors, and academic users to develop their products as designers and manufacturers. Nevertheless, to gain more effective and sustainable production, resource management with controlling appropriate print parameters for BMD part’s performance as well as manufacturing time and cost should be considered, especially for small manufacturers. Since BMD opens up a wide range of applications for AM parts with enclosures, it is important to optimize the internal lattice structure infill that enables specific strength and light weight. In general, a relation between the performance and the amount of material used should be optimized. The operating time and cost related to the amount of material used in the process are significant variables for sustainable management. An indicator assessing both the performance and sustainability of AM production should be utilized to achieve functional AM parts with optimal time and cost.

To the best of our knowledge, there is no existing report on the relationship between the performance and sustainability of AM products using BMD, which is vital for establishing sustainable production. This study aspires to investigate the effects of print parameters on the mechanical performance and sustainability of metal AM specimens with the BMD technique. The specimens with two sets of print parameters, i.e., infill density and pattern orientation, are tested using a tensile test to determine the mechanical performance. The relationship between the performance (ultimate tensile strength) and sustainability (manufacturing time and cost) of the specimens with varied infill densities is defined as an index for sustainable products. This paper provides a framework for end-users and prosumers to select appropriate print parameters for their AM products based on the index related to performance and sustainability. Moreover, although AM is named a sustainable manufacturing process, there are no explicit manners to indicate the sustainability level of AM products. The index in this study is consequently intended to propose a way to assess the sustainability of AM products.

## Results and discussion

### AM specimens designed for estimating the performance

**Figure 1:**
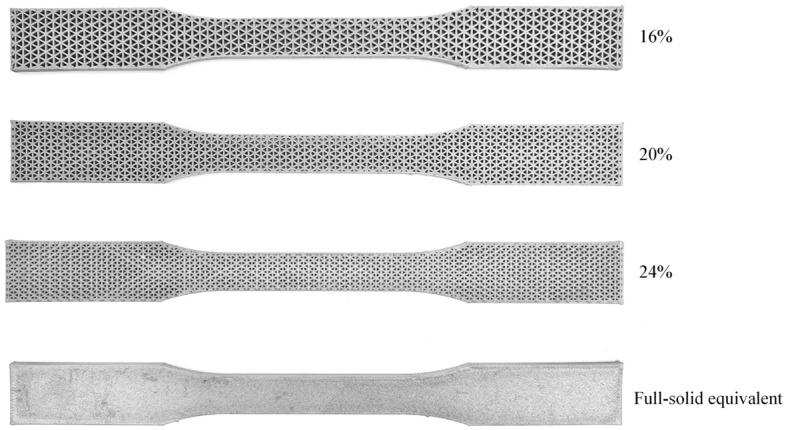
AM specimens with different infill densities.

In this study, tensile testing is performed to evaluate the mechanical properties of AM specimens with the BMD technique. The specimens, made of 17-4 PH stainless steel, were prepared for the test based on the ASTM standard with two print parameters considered. The first considered parameter is the infill density of AM specimens, which can be varied by adjusting the print parameter setting of the specimens through a DM’s web browser. Four infill densities are considered: 16%, 20%, 24%, and full-solid equivalent, as shown in Fig. 1. Note that a triangular pattern is a default infill pattern of the printer, and the density can be varied by modifying the sizes of the triangular unit cells.

**Figure 2:**
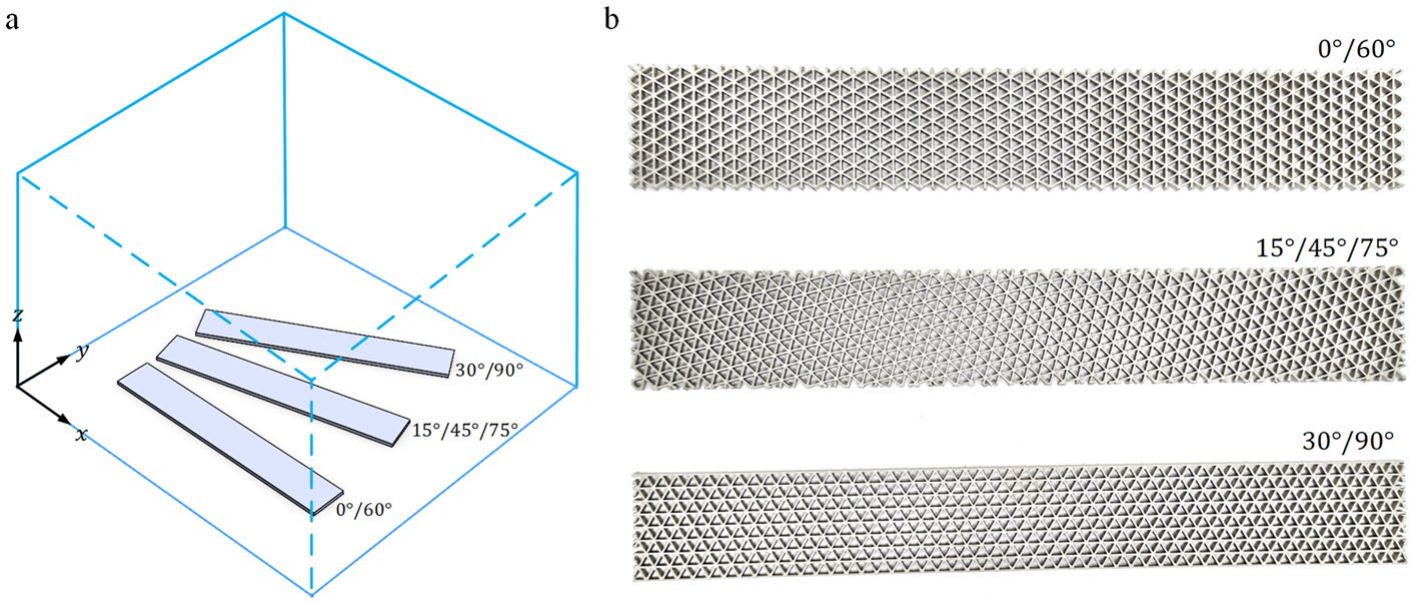
(**a**) Raster directions on the $x-y$ plane of the print platform. (**b**) The AM specimens with different infill pattern orientations.

The second considered parameter is the infill pattern orientation, laid on the $x-y$ plane, as shown in Fig. 2a. The varied orientations were set by adjusting the raster direction on the horizontal plane relative to the print platform to obtain specimens with capable stiffness^21^. Three different pattern directions are considered, i.e., the pattern with raster angles of 0, 15, and 30 degrees, as shown in Fig. 2b. For raster angles between 0 to 90 degrees, infill pattern orientations with 0, 15, and 30 degrees yield the same as those with 60, 45/75, and 90, respectively. Moreover, the infill patterns with different orientations will repeat the same patterns every 90 degrees. Consequently, in this study, the raster angles of 0/60, 15/45/75, and 30/90 are used to represent the perpendicular angles in other quarters. In this investigation, infill specimens without sidewalls are used for all samples. Note that the specimen dimensions are slightly different because they are designed to have eight complete unit cells along the short edges and the same density.

### Mechanical properties of infill specimens

The Young's modulus and ultimate tensile strength are the mechanical properties of the AM specimens considered in this study. The detail of data collection is in the section of the experimental design.

For the investigation of the specimens with different infill densities, the weights of each infill specimen in Fig. 1 are measured to compute their relative densities, defined as the ratio between the average weight of the infill specimen and its full-solid counterpart. The average values of the relative density, Young’s modulus, and ultimate strength are presented in Table 1.

**Table 1:** Average values of Young’s modulus and ultimate strength of the specimens with different infill densities.

Infill density (%)	Relative density (%)		Young’s modulus (GPa)		Ultimate strength (MPa)	
	Mean	SD	Mean	SD	Mean	SD
16	50.678	0.020	34.018	0.761	136.766	13.835
20	62.183	0.032	45.930	0.580	196.366	11.572
24	73.597	0.033	60.650	0.972	271.968	5.112
Full-solid equivalent	–	–	159.287	2.044	852.239	7.729

According to a datasheet provided by DM, the Young’s modulus and ultimate strength of 17-4 PH stainless steel specimens fabricated by the 3D metal printer are 195 GPa and 1042 MPa, respectively^29^. With the same test standard, the Young’s modulus and ultimate strength obtained in this study are approximately 81.69% and 81.79% of those in the datasheet, respectively. The discrepancy can occur due to various factors in the manufacturing processes. For this reason, an experimental study for a specific printing context is vital for assessing the mechanical properties of the AM parts.

The weight efficiency of the infill specimens with the considered stiffnesses, including Young’s modulus and ultimate strength, is presented as the normalized values in Table 2. These values were computed from Eq. (2) and used to compare the efficiency among infill specimens with different densities. The results show that the greater the infill density is, the more efficient the specimens. Nevertheless, this behavior is not linear because the weight efficiency rapidly increases when the specimen approaches being full-solid. Thus, the full-solid specimen is the best choice if only strength is the primary consideration. On the other hand, if weight or cost is considered as an additional need, the optimal condition for AM parts considering both strength and weight or cost is needed, discussed in the sustainability aspect in the next section.

**Table 2:** Normalized values of Young’s modulus and ultimate strength for evaluating the efficiency of the infill specimens.

Infill density (%)	Weight efficiency	
	Young’s modulus	Ultimate strength
16	0.421	0.317
20	0.464	0.371
24	0.517	0.434
Full-solid equivalent	1.000	1.000

For the investigation of the specimens with different pattern orientations, an infill density of 12% was used for all samples, of which the relative density measured was 36.12%. The average values of Young’s modulus and ultimate strength are presented in Table 3.

**Table 3:** Average values of Young’s modulus and ultimate strength of the specimens with different pattern orientations.

Pattern orientation (degree)	Young’s modulus (GPa)		Ultimate strength (MPa)	
	Mean	SD	Mean	SD
0/60	23.847	1.006	61.419	3.897
15/45/75	22.343	0.268	41.902	2.557
30/90	23.474	0.612	49.394	1.066

It can be seen that the pattern orientation almost does not affect the Young’s modulus of the specimens. For this reason, the specimens can be printed in any direction on the $x-y$ plane and yield the same load capacity throughout the elastic region. In contrast, applied load directions are significant for the specimen’s strength because the fluctuation of the ultimate strength shows during the change of the pattern orientations. The parts with the orientation of 0/60 degrees show higher ultimate strength than those with the orientations of 15/45/75 and 30/90 degrees (1.47 and 1.30 times higher, respectively). The AM parts with a pattern orientation of 0/60 degrees should be considered in the print parameter design for carrying loads to ensure that the parts yield maximum in-plane ultimate strength.

### Experimental validation

Since an infill specimen with a triangular pattern can be treated as a lattice structure with triangular unit cells, the effective Young’s modulus of such a lattice can be predicted using the closed form obtained from the literature^30^. The specimen with the unenclosed sidewalls and pattern orientation of 0/60 degrees, as shown in Fig. 2, is concordant with a lattice structure, and the Young’s modulus can be computed using Eq. (1). In the validation, the value of the Young’s modulus obtained from the experiment in Table 3 is compared to that calculated from Eq. (1), which is equal to 23.107 GPa. Good agreement is observed with an error of 3.10%.

To compare the results in this study with related works, the weight efficiency of the infill specimen with the orientation of 0/60 degrees (see Fig. 2) was compared with that obtained from the literature. The weight efficiency can be computed using Eq. (2) using Young's modulus as the considered stiffness. The results obtained from this study and the related works are 0.35, 0.34^30^, 0.33^31^, and 0.35^32^. These results are well consistent and confirm the experimental results in this study.

**Figure 3:**
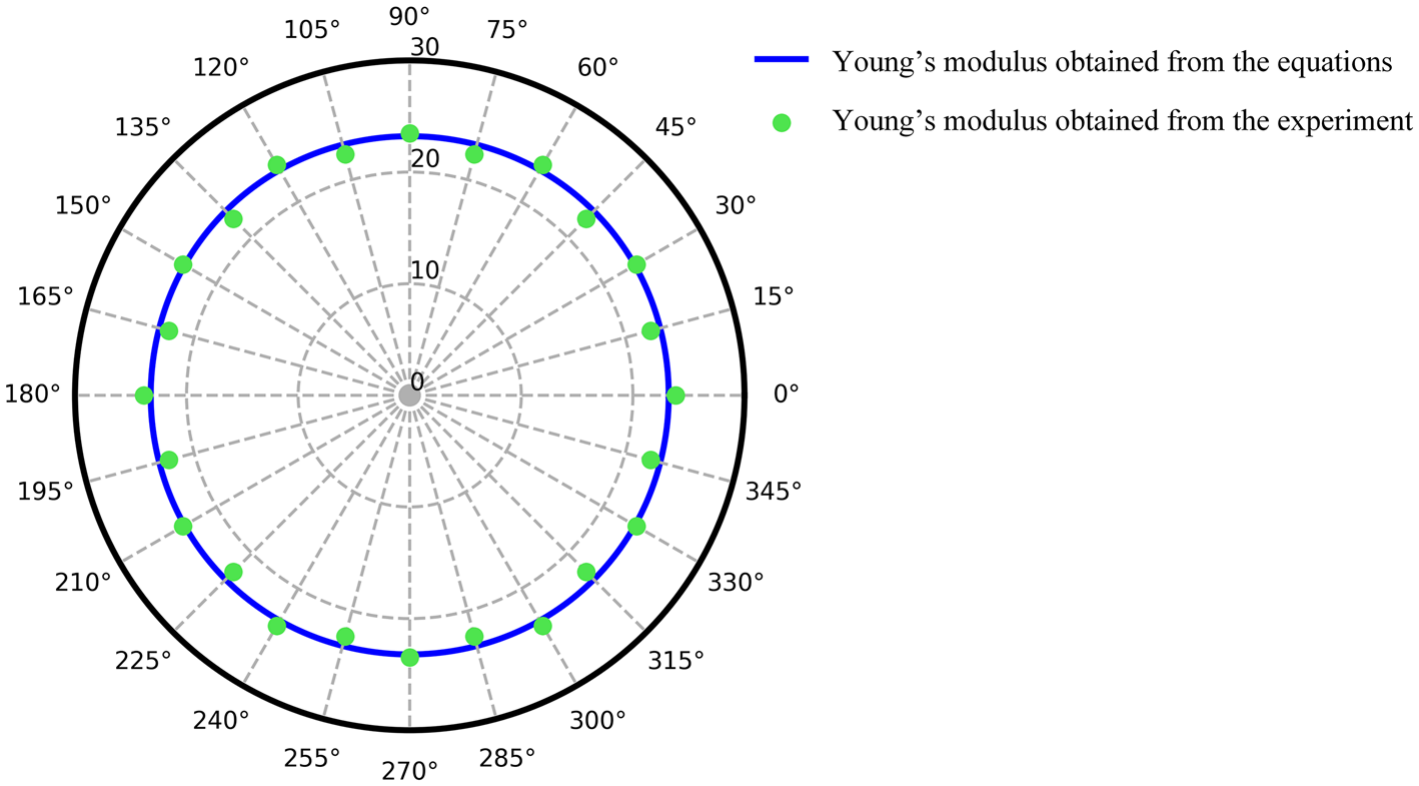
Isotropic polar plot of the infill specimens with different pattern orientations.

In addition, by substituting Eq. (1) in Eqs. (3) and (4), the in-plane Young’s modulus of the specimens in other directions can be computed. The Young’s modulus results of the specimens with different orientations ranging from $0$ to 360 degrees, obtained from the formulas and experiment, are plotted in Fig. 3. The transformation of the Young’s modulus, obtained from Eqs. (3) and (4), is illustrated as the blue line, while the experimental results of the Young’s modulus of the specimen with the different pattern orientations are presented as the green points. It can be seen in the isotropic polar plot that both results are similar for all corresponding directions with an absolute error of less than 4.00%.

### Sustainability aspect

Environmental, societal, and economic aspects are general factors used to assess the sustainability of a product. AM has been proven from many studies to be an eco-friendly manufacturing process compared to conventional processes^5,33–35^. AM yields direct effects regarding better environmental impacts, such as particle pollution, contamination, and hazardous wastes. At the same time, it also yields indirect environmental impacts by providing a reduction of CO_2_ emissions from less usage of raw material in the process. These impacts lead to societal improvement with better quality of life and health benefits for producers. The societal aspect also impacts the rise of customers interested in designing and producing, called prosumers, since AM enables them to achieve sustainability goals and develop new business opportunities^5,36–38^. As mentioned in the introduction, these advantages can be more efficient in AM technology using BMD, which provides more accessible, safe, and effective metal part manufacturing.

From an economic perspective, the potential increased involvement of prosumers fuels the circular economy from emerging new business models with up-to-date manufacturing and logistics, e.g., on-demand production and instant supply. In addition, AM with BMD can be proposed in terms of time-saving and cost-effective production resulting from minimizing the metal used in the process. The less material used, the less manufacturing time and the lower the costs. Thus, selecting an appropriate infill density for parts, matching their application, is essential because the material density is directly related to performance, as discussed earlier. Table 4 presents the time and costs for fabricating AM specimens with different infill densities using BMD. The expenses were classified into direct and indirect costs and are considered in the cost model; the labor cost is not considered because it is negligible in the BMD process. The whole time of the manufacturing process, i.e., printing, debinding, and sintering, is included in the operating time in terms of hours.

**Table 4:** Time and costs in the BMD manufacturing process of the AM specimens.

Infill density (%)	Material cost ($)	Direct cost ($)	Indirect cost ($)	Estimated total cost ($)	Operating time (h)
16	10.21	21.69	317.58	339.28	69.00
20	11.88	23.69	336.97	360.67	73.12
24	13.55	25.66	351.66	377.32	76.26
Full-solid equivalent	17.40	30.19	407.37	437.56	87.98

**Figure 4:**
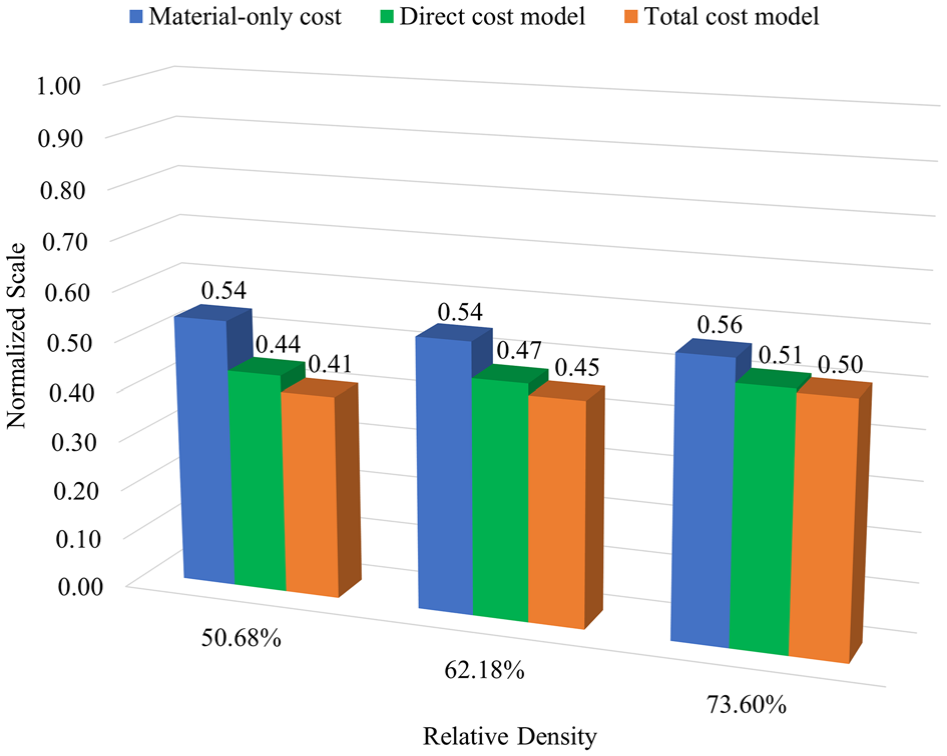
The weight efficiency against the normalized values of the operating cost.

The material cost, including only the metal and interface costs, is a part of the direct cost, which also considers the costs of electricity and consumables, e.g., debind fluid and gases. Most of the expenditure is from the indirect cost, which is approximately 93% to 94% of the total cost. As shown in Table 4, the time and costs increase according to the amount of material used, related to the percentage of infill density of the specimens. Therefore, adjusting the time and cost to be optimal for the desired weight efficiency is a way to run a sustainable process. This study defines the relationship between the weight efficiency and normalized sustainable parameter(s) as an index for sustainable products. The weight efficiency can be computed using Eq. (2), which is defined as ${e}_{UTS}$ when the ultimate strength is considered. Meanwhile, the operating cost and manufacturing time are treated as sustainable parameters. A normalized sustainable parameter can be obtained from a ratio between a considered sustainable parameter of each infill specimen and that of the full-solid counterparts. For example, the normalized operating cost can be obtained from the ratio between the cost of the infill specimens and such cost of the full-solid counterparts, defined as ${\widehat{C}}_{i}={C}_{i}^{\text{infill}}/{C}_{i}^{\text{solid}}$, where $i$ denotes a considered cost, i.e., material-only cost, direct cost, and total cost. The index can be consequently obtained by ${e}_{UTS}/{\widehat{C}}_{i}$, which can be adjusted for various economic aspects by considering different focused cost models $i$. Note that, since the indirect costs are close to the total expenses and both yield the same index, only the results of the total costs are presented. As shown in Fig. 4, the index values, where $i$ denotes material-only cost, direct cost, and total cost, are presented as blue, green, and orange, respectively. The assessment from the perspectives of performance, weight, and cost is included in the index model as the normalized values. A higher index value implies that AM products yield better performance against sustainability.

Similar index values are observed for all considered infill specimens, focusing on the material-only cost. It is implied that infill density has less effect when considering the weight efficiency versus the normalized cost of only materials used, especially for the specimens with 50.68% and 62.18% relative densities. In contrast, focusing on the direct and total costs, the specimens with a higher infill density yield a better index. It is shown that even though the specimens with higher infill density have more expenses, they give good results in terms of performance and sustainability.

**Figure 5:**
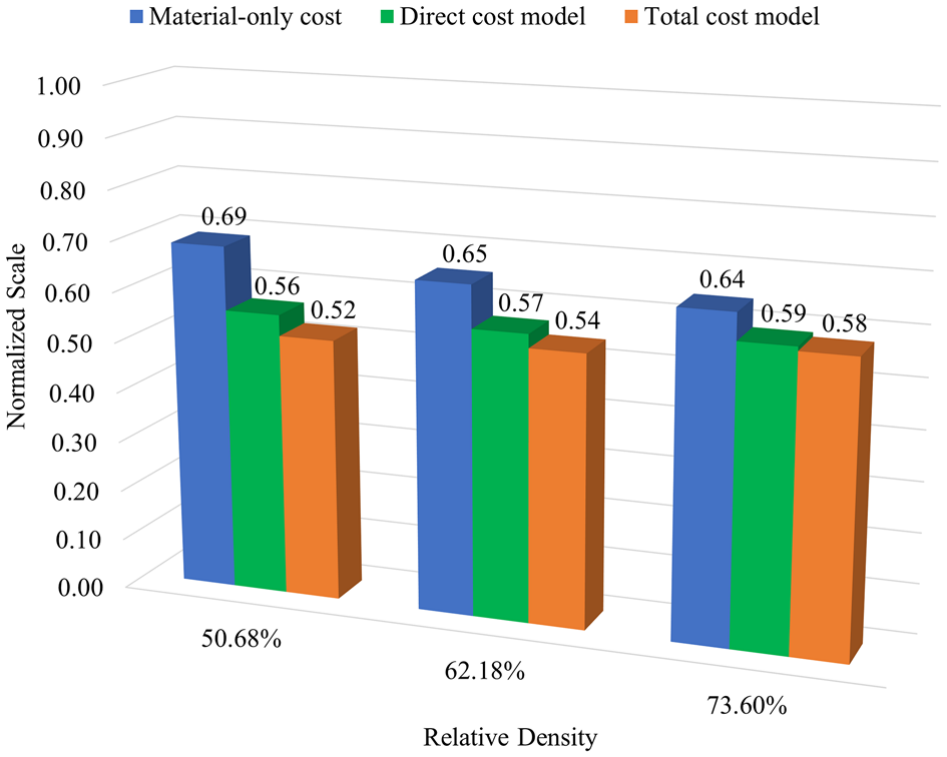
The weight efficiency against the normalized values of the operating cost and time.

In addition, to modeling a more comprehensive index from the sustainability point of view, the time used in the manufacturing process can be included in the index model. The index adding the manufacturing time can be obtained by ${e}_{UTS}/({\widehat{C}}_{i}\times {\widehat{T}}_{P})$, where ${\widehat{T}}_{P}$ denotes the normalized values of the whole processing time. The assessment in terms of performance, weight, cost, and time aspects is included in this index, as shown in Fig. 5.

It can be seen from the model in Fig. 5 that the index values increase when the manufacturing time is considered additionally. Although adding more sustainable parameters in the model, most AM parts with a higher infill density are still better than those with a lower infill density. However, when the material-only cost is considered, the parts with a lower infill density can yield a higher index than those with a higher infill density. Since the sensitivity of the index varies depending on sustainable parameters, considering more sustainable parameters (e.g., energy consumption and CO_2_ emissions from the raw material used) and specific cost models (e.g., material-only cost) can significantly make the parts with a lower infill density have more impact from the point of view of performance versus sustainability. Note that considering specific cost models like the material-only cost is practicable for the assessment because most total costs consist of committed fixed costs like indirect costs, which are fixed obligations of the business.

In fact, the infill parts cannot match the full-solid parts in terms of the capability of the in-plane stiffness. This study intends to propose an indicator to assess the AM parts with an infill density not only concerning the performance aspect but also the sustainability aspect. The relationship between mechanical properties and the costs and time, which is presented in terms of the ratio of weight efficiency and normalized sustainable parameters, can be used to assess the degree of sustainability of an AM product initially. The higher value of the index demonstrates the product is preferable in terms of performance versus sustainability.

Furthermore, this study focuses on AM products’ performance and sustainability points of view. Only Young's modulus and ultimate strength are considered for the performance aspect while manufacturing cost and time are considered for the sustainability aspect. The suggestions for future studies are that, in the performance aspect, other stiffness, such as bending and fatigue, should be further studied to cover more performance. In the sustainability aspect, more sustainable parameters can be considered in the model, such as the energy and environment aspects. Moreover, this study considers only the manufacturing costs of AM products. An analysis related to the gain or loss from an investment may be taken into account further to cover the commercial aspect.

## Conclusion

The 17-4 PH stainless steel specimens fabricated by AM using the BMD technique were tested by tensile testing to investigate the effects of the print parameters on the performance and sustainability. From the performance point of view, the AM specimens with two sets of print parameters, i.e., the specimens with different infill densities and the specimens with varying pattern orientations, were tested to obtain the Young’s modulus and ultimate tensile strength. As expected, the specimens with a higher infill density yield better mechanical properties. The in-plane pattern orientations have a little effect on the Young’s modulus of the infill specimens with the triangular pattern, but there are differences in the ultimate strength. The specimens with a pattern orientation of 0/60 degrees yield the best ultimate strength.

From the sustainability point of view, the manufacturing time and cost of the specimens with different infill densities are considered sustainable parameters, while the ultimate strength is selected to be the considered performance. The relationship between the ultimate strength of an infill specimen and its normalized weight density is defined as the weight efficiency of the specimen. The index for assessing an AM part in terms of performance versus sustainability is characterized by the weight efficiency versus the normalized sustainable parameter(s). Two assessment models are proposed in this study: (1) the weight efficiency versus the normalized operating cost and (2) the weight efficiency versus the normalized operating cost and time. The material-only, direct, and total costs are classified as the cost model options. The results show that considering the material-only cost (a limit case), the infill specimens with a lower density yield a comparable index to those with a higher density in the first model, while, in the second model, the infill specimens with a lower density yield a better index than those with a higher density. In addition to these cases, the infill specimens with a higher density have the best index for all cases. Note that the performance of AM specimens in this study was investigated only for in-plane loading. Other implementations, such as bending and fatigue, may give different results.

As shown by the results of this study, it can be concluded that the in-plane pattern orientation (horizontal plane) hardly significantly affects the Young’s modulus of the infill parts with the triangular pattern. The Young’s modulus of the infill parts is not a linear function of the infill density. The parts with a higher infill density yield higher weight efficiency of in-plane stiffness than those with a lower infill density. When considering performance versus sustainability, most parts with a higher infill density yield a higher index than those with a lower infill density. However, the assumption of sustainable parameters can significantly change the index. The index can be used to evaluate AM parts in terms of stiffness against sustainability.

## Methods

### Effective properties of lattice structures

For an inhomogeneous material, if its inhomogeneities are uniformly distributed and considerably smaller than the full size of the material, at least stochastically, the material can be considered a homogeneous material with effective properties. The material is considered theoretically extended to become an infinite material in determining its effective properties. It is subjected to uniform boundary conditions that yield uniform stresses and strains in a homogeneous material at its far-field boundary. The resulting relations between the average stresses and average strains can then be used to define the effective properties of the material^39,40^.

An inhomogeneous material in which the inhomogeneities are distributed periodically can be considered a periodic material, also known as a lattice structure. If a lattice structure consists of a substantial number of periodic unit cells, the effective properties can be determined^41,42^. In manufactured lattice structures, such unit-cell patterns are used to help reduce the amounts of raw materials used (compared to solid material) while retaining the desired properties matching their applications. Different periodic patterns or unit-cell patterns result in the various mechanical behaviors of lattice structures.

**Figure 6:**
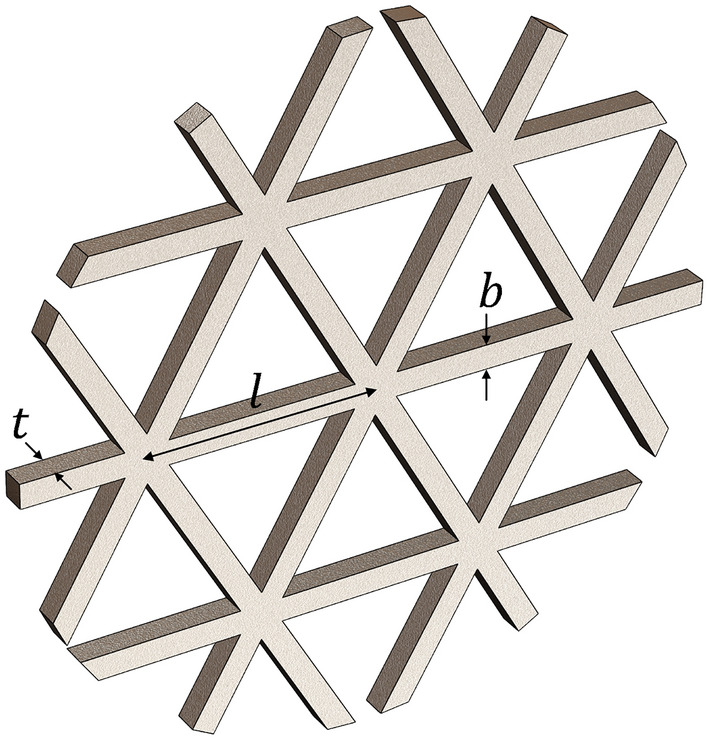
A lattice structure with triangular unit cells and its strut dimensions.

A 2D lattice structure is often used for an AM product to reduce raw materials and manufacturing costs by controlling the densities of their infill patterns. A 2D periodic pattern is generally used for the infill pattern of an AM part, especially a triangular pattern. In this study, AM specimens with triangular infill patterns are treated as 2D lattice structures with triangular unit cells. The material behavior of the triangular infill specimens can then be obtained by considering the effective elastic properties of a lattice structure with triangular unit cells. Figure 6 shows a lattice structure with triangular unit cells, where $l$ denotes the characteristic length of the unit cells and $b$ and $t$ denote the width and thickness, respectively. These significant parameters can be used to compute the effective Young’s modulus of lattice structures with triangular unit cells obtained from the literature^30^, i.e.,

1 $${E}_{x}^{*}={E}_{y}^{*}=E\overline{\rho }\left(\frac{{l}^{2}+{b}^{2}}{3{l}^{2}+{b}^{2}}\right)$$

where ${E}_{x}^{*}={E}_{y}^{*}$ denotes the effective Young’s modulus of the infill specimens and $E$ denotes the Young’s modulus of the base material. In addition, $\overline{\rho }$ denotes the relative density of the lattice structure with triangular unit cells that is equal to $2\sqrt{3}b/l$.

### Weight efficiency of a lattice structure

An infill density is used in an AM specimen to reduce its weight. When the specimen stiffness is among the leading design targets, the ratio of the specimen stiffness to its weight density can be considered to evaluate weight efficiency. To assess the efficiency of an infill specimen compared to its full-solid counterpart, the ratio between the considered stiffness per weight density of the specimen and the stiffness per weight density of a full-solid specimen having the same dimensions is defined as weight efficiency of the specimen, i.e.,

2 $${e}_{S}=\frac{\left(\frac{{S}^{*}}{{\rho }^{*}}\right)}{\left(\frac{S}{\rho }\right)}=\frac{1}{\overline{\rho }}\frac{{S}^{*}}{S}$$

where ${S}^{*}$ and $S$ are the considered stiffnesses of the infill specimen and the full-solid counterpart, respectively. In addition, $\overline{\rho }$ is the relative density, which is, in fact, the ratio between the weight density of the infill specimen and the weight density of a full-solid specimen having the same dimensions, i.e., $\overline{\rho } = {{\rho^{*} } \mathord{\left/ {\vphantom {{\rho^{*} } \rho }} \right. \kern-\nulldelimiterspace} \rho }$.

### Material properties in other directions

**Figure 7:**
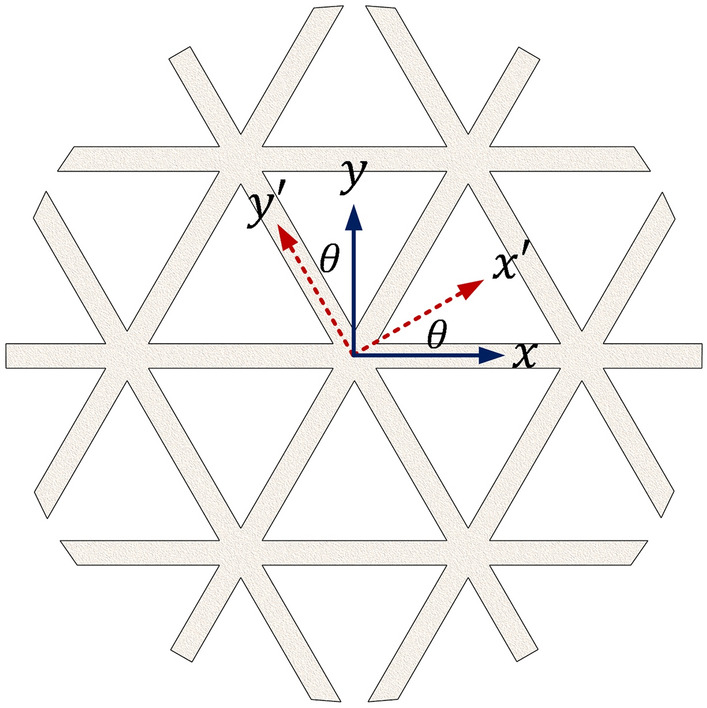
Original and rotating coordinate systems.

The effect of the load orientations on 2D lattice structures on a $x-y$ plane is investigated to validate lattice structures’ properties in different rotating directions. For orthotropic materials, the transformation of mechanical properties from the $x-y$ coordinate system to the ${x}^{^{\prime}}-{y}^{^{\prime}}$ coordinate system is illustrated in Fig. 7.

The transformation of Young’s modulus between the two coordinate systems, in which the stiffness and compliance matrices are symmetric, can be written as^43^

3 $${E}_{{x}^{^{\prime}}}=\frac{{E}_{x}}{{m}^{4}+\left(\frac{{E}_{x}}{{G}_{xy}}-2{\nu }_{xy}\right){n}^{2}{m}^{2}+\frac{{E}_{x}}{{E}_{y}}{n}^{4}}$$

4 $${E}_{{y}^{^{\prime}}}=\frac{{E}_{y}}{{m}^{4}+\left(\frac{{E}_{y}}{{G}_{xy}}-2{\nu }_{yx}\right){n}^{2}{m}^{2}+\frac{{E}_{y}}{{E}_{x}}{n}^{4}}$$

where ${E}_{x}$ and ${E}_{y}$ denote the Young’s modulus in the $x-y$ coordinate system, while ${E}_{{x}^{^{\prime}}}$ and ${E}_{{y}^{^{\prime}}}$ denote Young’s modulus in the $x{^{\prime}}-y{^{\prime}}$ coordinate system. In addition, ${G}_{xy}$ denotes the shear modulus, and ${\nu }_{xy}={\nu }_{yx}$ is Poisson’s ratio. Here, $m=cos\theta$ and $n=sin\theta$ are defined for the transformation of geometrical points.

### Experimental design

The mechanical properties of AM parts can be experimentally obtained by a tensile test based on the ASTM E8/E8M standard. Since an AM part is treated as a lattice structure constructed by many unit cells inside, its specimen should be designed as a homogeneous material with effective properties to ensure that it yields an exact mechanical response^44,45^. Other print parameters affecting its mechanical properties can be studied when ensuring that a specimen can be considered a homogeneous material. In this paper, two significant print parameters have been emphasized, i.e., infill density and pattern orientation, to investigate the effects of print parameters of AM technology on material behavior and manufacturing costs. Four relative densities are considered for the AM specimens, including 50.68%, 62.18%, 73.60%, and 100% (equivalent) of the full-solid specimen, as shown in Fig. 1. Three pattern orientations of the triangular unit cells are considered, including the pattern orientation rotated $0^\circ$, $15^\circ$, and $30^\circ$, as shown in Fig. 2. Since the rotation of $0^\circ$, $15^\circ$, and $30^\circ$ yields symmetrical geometry with $60^\circ$, $45^\circ /75^\circ$, and $90^\circ$, respectively, the infill pattern orientations considered are then sufficient for the investigation in this study.

**Figure 8:**
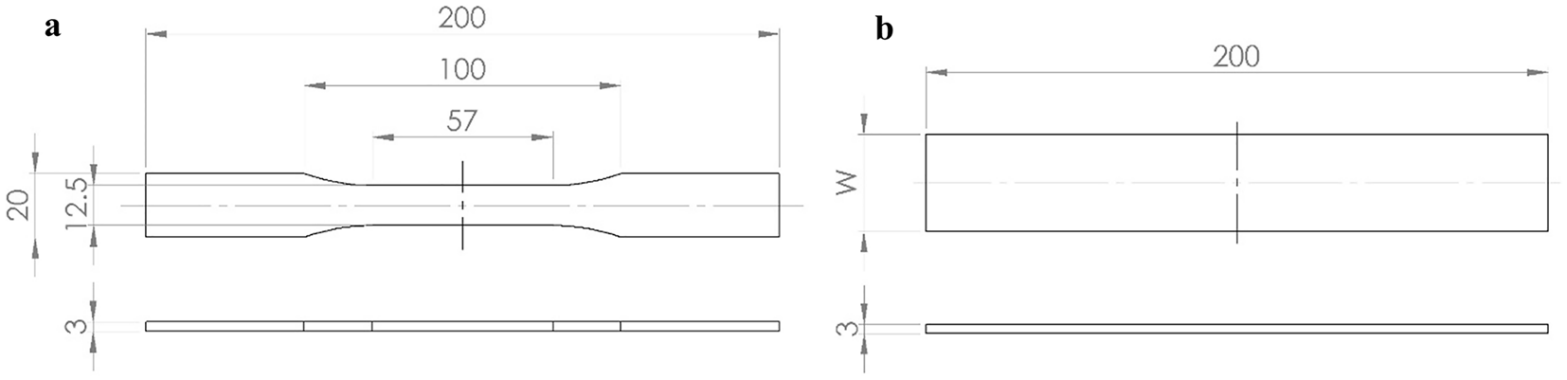
The geometry and dimension of a specimen according to the ASTM E8/E8M standard.

All specimens, made of 17-4PH stainless steel, were fabricated using BMD technology by a Studio System Desktop Metal 3D printer. The specimens with infill density using triangular pattern were created without top and bottom wall thicknesses to investigate the infill density effect. The geometry and dimension of the specimens were considered according to the ASTM E8/E8M standard, as shown in Fig. 8a. The infill densities were varied by changing $l$ while maintaining the rectangular strut’s cross-section (see Fig. 6). The infill densities are also presented as relative densities, which is a ratio between the weight of infill specimens and the full-solid one, as shown in Table 1. For the specimens used to investigate the pattern orientation effect, the specimens with a rectangular shape were designed based on the ASTM E8/E8M standard, as shown in Fig. 8b, where $\mathrm{W}$ can be adjusted to obtain complete unit cells along the short edge. In the investigation, the samples with 36.12% relative densities were used for each pattern orientation. Note that five samples were examined for a set of specimens in each parameter type. The reliable results of at least three samples were collected to compute average values of Young’s modulus and ultimate strength.

According to the ASTM E8/E8M standard, a tensile test was performed using an Instron 8802 universal testing machine to obtain the Young’s modulus and ultimate tensile strength of the 17-4 PH stainless steel specimens. The tensile tests were operated in displacement control with 1.0 mm/min speed before the yield point and 5.0 mm/min speed after the yield point. An extensometer was used to measure strain in the elastic region and then removed after the yield point.

### Cost estimation for AM production

A unit cost model was used to estimate the total costs of AM products during the manufacturing process. A general equation of the total cost model ${C}_{T}$ can be written as

5 $${C}_{T}={C}_{\text{Direct}}+{\dot{C}}_{\text{Indirect}}\left({T}_{P}\right)+{\dot{C}}_{\text{Labor}}\left({T}_{L}\right)$$

where ${C}_{\text{Direct}}$ denotes the estimated cost incurred for raw material, interface, consumables, and electricity, while ${\dot{C}}_{\text{Indirect}}$ and ${T}_{P}$ denote the cost rate of the machine operation and processing time. In addition, ${\dot{C}}_{\text{Labor}}$ and ${T}_{L}$ denote the labor rate and the labor duration.

Summation of element costs, i.e., direct, indirect, and labor costs related to the printing activity, reflects the cost of the primary BMD process. The direct cost estimate captures all expenditures for materials fed into the system (raw material and interface), energy consumed, and consumables (build sheet, debind fluid, gas, etc.). The indirect cost rate represents the main total machine costs of depreciation and maintenance, which can be obtained by

6 $${\dot{C}}_{\text{Indirect}}=\left(\frac{\text{Machine purchase}}{\text{Depreciation period}}+\text{Estimated maintenance} \right)\times \frac{1}{\text{Annual operating time}}$$

In this study, the printing machine was estimated to operate for a ten-year period, while the annual maintenance cost was estimated at 15% to 20% of the yearly machine cost, depending on each machine type. In addition, the annual operating time was set to 4,000 h. Equation (6) was used to compute the total machine cost rate in Table 5. The labor cost estimate describes the costs waging for an AM production worker. Table 5 shows the elements of the total cost model throughout the manufacturing process. Note that all estimated costs are modeled based on the machine owner context.

**Table 5:** Elements of the unit cost model.

Cost description	Price ($)	Unit
**Direct cost**		
Raw material	190.212	Kilogram
Interface	1917.334	Kilogram
Debind fluid	159.778	Lite
Electricity	0.132	Kilowatt-hour
**Indirect cost**		
Total machine cost rate	4.690	Hour
**Labor cost**		
Labor rate	1.213	Hour

## Data Availability

The datasets used and/or analysed during the current study available from the corresponding author on reasonable request.
